# Extension of the Flory-Rehner Theory of Swelling to an Anisotropic Polymer System

**DOI:** 10.6028/jres.065A.051

**Published:** 1961-12-01

**Authors:** Stephen D. Bruck

## Abstract

The Flory-Reimer theory for isotropic swelling of rubber crosslinked in the dry state is extended to an anisotropic system crosslinked in the dry, oriented state. The new parameters introduced into the equation can be readily determined from dimensional changes of the fiber in a suitable solvent using a photomicrographic technique. Unlike other methods, such as the cathetometric and weight methods, this technique enables the attainment of swelling equilibrium usually within 30 minutes. Good agreement is obtained between the equivalents of crosslinks calculated from chemical analyses and from swelling measurements, respectively.

## 1. Introduction

An important objective in the proper evaluation of network structures is the determination of swelling equilibrium volume ratio $q_{m}=\frac{V}{V_{0}}$, and the calculation of the average molecular weight between crosslinks $\left({\bar{M}}_{c}\right)$. There are two classical methods for the determination of the swelling equilibrium volume ratios: (a) the weight method, and (b) the linear method with a cathetometer. Using the weight method one obtains the ratio of the weight of the swollen network at equilibrium to the weight of the unswollen crosslinked polymer, and from a knowledge of the densities of the solvent and polymer, one can calculate $q_{m}=\frac{V}{V_{0}},$ (where *V* = volume of the swollen network at equilibrium and *V*_0_ = volume of the unswollen crosslinked network). Using the linear method one measures the length of the fiber at swelling equilibrium (*L*), and the length of the original fiber in the dry state (*L*_0_). The equilibrium volume swelling ratio, *q_m_*, will then be equal to ${\left(\frac{L}{L_{0}}\right)}^{3}$ if the system is isotropic.

With relatively highly crosslinked networks, especially in case of oriented fibers, neither of the above-mentioned methods is practical. Swelling equilibrium in this case is reached very slowly (up to several weeks) because of surface effects of the fiber. Also, because the system is anisotropic, *q_m_*, is no longer equal to (*L/L*_0_)^3^.

It is the object of this paper to present a modification of the Flory-Rehner theory of swelling and to describe a photomicrographic technique for the rapid determination of swelling equilibrium volume ratios in crosslinked fibers. The Flory-Rehner theory for isotropic swelling of rubber crosslinked in the dry state is extended to an anisotropic system cross-linked in the dry, oriented state. It is shown that good agreement is obtained between the equivalents of crosslinks calculated from chemical analyses and from swelling measurements, respectively.

### Modification of the Flory-Rehner Theory of Swelling

Although the Flory-Rehner treatment [1, 2]^1^ of the isotropic swelling of rubber crosslinked in the dry state has been found to apply to radiation crosslinked, essentially *isotropic* polyamide films [3], *oriented (hence anistropic) structures* represent a difficulty. The Flory-Rehner expression for the isotroplc swelling of rubber is:

$$\begin{array}{l} -[\ln\;(1-v_{2m})+v_{2m}+\chi_{1}v_{2m}^{2}]=\\ \;\frac{V_{1}}{\bar{v}M_{c}}\left(1-\frac{2M_{c}}{M}\right)\left(v_{2m}^{1/3}-\frac{v_{2m}}{2}\right) \end{array}$$ (1a)

where *v*_2_*_m_*= 1/*q_m_*, which is the ratio of the volume of the unswollen network (*V*_0_) to the volume of the swollen network at equilibrium (*V*); *V*_1_ = molar volume of solvent; $\bar{v}$ = specific volume of swollen polymer; ${\bar{M}}_{c}$ = number average molecular weight between crosslinks; *M*= primary number average molecular weight of polymer (before crosslinking); χ_1_ = interaction parameter which is a measure of the interaction energy of solvent molecules with polymer.

According to Flory [4] the term $1/v_{2m}=\alpha_{s}^{3}$, where *α_s_* is the linear deformation factor; hence the above equation may be rewritten as follows:

$$-[\ln\;(1-v_{2m})+v_{2m}+\chi_{1}v_{2m}^{2}]=\frac{V_{1}}{2\bar{v}M_{c}}\left(1-\frac{2{\bar{M}}_{c}}{M}\right)\left(\frac{2}{\alpha_{s}}-\frac{1}{\alpha_{s}^{3}}\right)$$ (1b)

Since in the case of crosslinked, *anisotropic* fibers swelling does not take place equally in three dimensions, the linear deformation factor, *α_s_*, must be so expressed as to identify the swelling components in the *x* and *y* directions. Hence the equilibrium volume swelling ratio for an anisotropic crosslinked fiber (2-dimensional isotropy where *α_x_≠α_y_=α_z_*) may be expressed as:

$$q_{m}=\frac{V}{V_{0}}=\frac{L\times D^{2}}{L_{0}\times D_{0}^{2}}$$ (2)

where *V* = volume of swollen structure at equilibrium; *V*_0_=volume of unswollen network; *L*=length of swollen structure at equilibrium; *D*=diameter of swollen structure at equilibrium; *L*_0_=length of unswollen structure; *D*_0_=diameter of unswollen structure.

In order to utilize the parameters *L, L*_0_, *D*, and *D*_0_, additional terms must be introduced into the original Flory-Rehner equation. The modified equation is derived using the equations in the Flory-Rehner treatment, as follows (see also references [1, 2, and 4]):

$$\Delta F=\Delta F_{m}+\Delta F_{el}$$ (3)

where Δ*F*= total free energy change involved in the mixing of pure solvent with the crosslinked network; Δ*F_m_*=free energy of mixing; Δ*F_el_*= elastic free energy.

The free energy change on mixing is:

$$\Delta F_{m}=\Delta H_{m}-T\Delta S_{m}=kT[n_{1}\;\ln\;v_{1}+\chi_{1}n_{1}v_{2}]$$ (4)

where *v*_1_ = volume fraction of pure solvent; *v*_2_= volume fraction of polymer; *n*_1_=number of solvent molecules.

Also the elastic free energy is:

$$\Delta F_{el}=\Delta H_{el}-T\Delta S_{el}$$ (5)

where Δ*S_el_* = change in entropy due to configurational change of the network; Δ*H_el_*=change of enthalpy of the network which approximately=0 because (by analogy with the deformation of rubber) the deformation process during swelling is assumed to occur without appreciable change in internal free energy of the network. From the statistical theory of rubber elasticity,

$$\Delta F_{el}=-T\Delta S_{el}=\frac{kTv_{e}}{2}[\alpha_{x}^{2}+\alpha_{y}^{2}+\alpha_{z}^{2}-3-\ln\;(\alpha_{x}\alpha_{y}\alpha_{z})]$$ (6)

where *α_y_*= linear deformation factor in *y* direction; *α_x_*=linear deformation factor in *x—*direction; *α_z_*= linear deformation factor in *z* direction; and *v_e_*= effective number of chains in the network.

Since in the case of two-dimensional isotropy, such as in crosslinked fibers, *α_x_≠α_y_=α_z_*, then, calling *α=α_y_=α_z_*

$$\Delta F_{el}=-T\Delta S_{el}=\frac{kTv_{e}}{2}[2\alpha^{2}+\alpha_{x}^{2}-3-\ln\;(\alpha^{2}\alpha_{x})].$$ (7)

The chemical potential of the solvent in the swollen network with two-dimensional isotropy (fibers) is:

$$\begin{array}{l} \mu_{1}-\mu_{1}^{0}=N{\left(\frac{\partial \Delta F_{m}}{\partial n_{1}}\right)}_{T,P}+\frac{N}{2}[\left(\frac{\partial (\Delta F_{el})}{\partial (\alpha )}\right)\left(\frac{\partial (\alpha )}{\partial (n_{1})}\right)\\ \;{+\left(\frac{\partial (\Delta F_{el})}{\partial (\alpha_{x})}\right)\left(\frac{\partial (\alpha_{x})}{\partial (n_{1})}\right)]}_{T,P} \end{array}$$ (8)

where *N*=Avogadro’s number. Evaluating the terms in eq (8) and noting that at equilibrium swelling $\mu_{1}=\mu_{1}^{0}$ and *v_2_=v_2m_* we get:

$$\begin{array}{l} -[\ln\;(1-v_{2m})+v_{2m}+\chi_{1}v_{2m}^{2}]\\ \;=\frac{V_{1}}{2\bar{v}M_{c}}\left(1-\frac{2M_{c}}{M}\right)\left(\frac{1}{\alpha_{x}}-\frac{1}{\alpha^{2}\alpha_{x}}+\frac{\alpha_{x}}{\alpha^{2}}\right) \end{array}$$ (9a)

or

$${\bar{M}}_{c}=\frac{\frac{V_{1}}{2\bar{v}}\left(\frac{1}{\alpha_{x}}-\frac{1}{\alpha^{2}\alpha_{x}}+\frac{\alpha_{x}}{\alpha^{2}}\right)}{-[\ln(1-v_{2m})+v_{2m}+\chi_{1}v_{2m}^{2}]+\frac{V_{1}}{\bar{v}M}\left(\frac{1}{\alpha_{x}}-\frac{1}{\alpha^{2}\alpha_{x}}+\frac{\alpha_{x}}{\alpha^{2}}\right)}$$ (9b)

In the above equation, $\alpha_{x}=\frac{L}{L_{0}}$ and $\alpha =\frac{D}{D_{0}}$.

## 2. Experimental Procedure

Experiments were conducted on a series of 7.8 Tex (60 denier)/32 filament nylon-6 (polycaprolactam) fibers, ${\bar{M}}_{n}$ (number average molecular weight) = 14,000 (from end-group analyses). Crosslinking was carried out with gaseous formaldehyde on the solid, oriented (drawn) fibers [5]. It can be shown that the total equivalents of amide groups in 10^6^ g of nylon-6 polymer is 8,850 (neglecting end-groups), which corresponds to a theoretical maximum of approximately 4,425 equivalents of crosslinkages if all nitrogen atoms (in crystalline and amorphous regions) were substituted. Since nylon-6 fiber is approximately 50 percent crystalline, there would be a maximum of approximately 2,212 equivalents of crosslinkages in the amorphous portions of 10^6^ g of polymer.

The values *L, L*_0_, *D*, and *D*_0_ are obtained from the dimensional changes of small segments (about 0.5 mm) of single filaments in a suitable solvent (such as *m*-cresol) using a microscope and a micrometer eyepiece or a photomicrographic assembly. A small segment of the yarn is placed on a microscope slide and onto the phase-microscope stage. The specimen is photographed using a Polaroid camera (or measured with a micrometer eyepiece) with a magnification of between 100× to 400× depending on the dimensions of the fiber. From the photographs the values *L, L*_0_, *D*, and *D*_0_ can then be readily measured.

**Figure 1:**
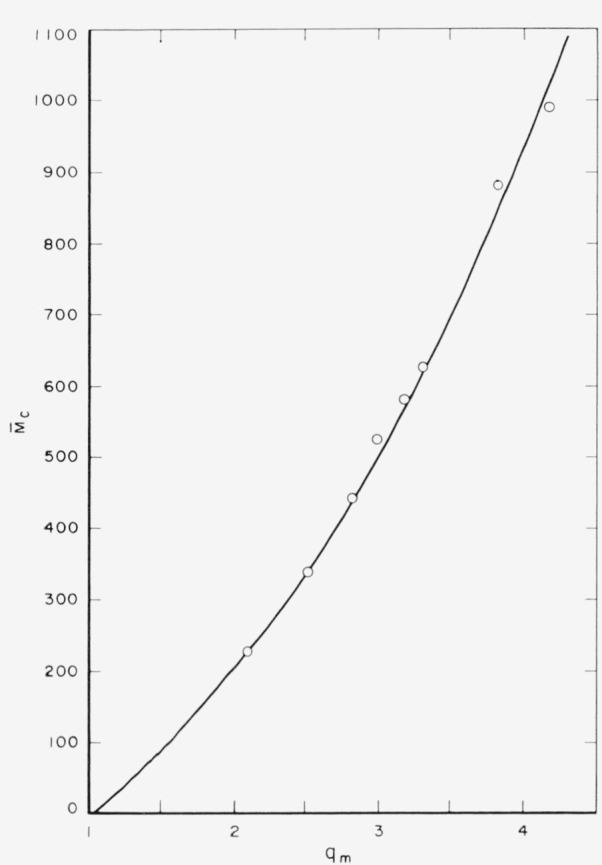
The relationship between ${\bar{M}}_{c}$ and *q_m_*.

**Figure 2:**
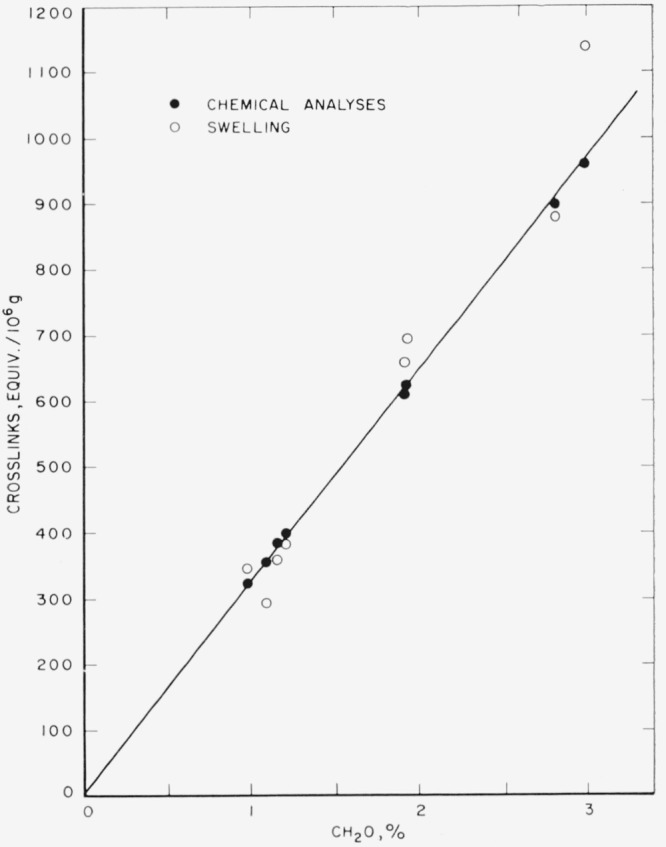
Correlation between percent formaldehyde and equivalent number of crosslinks.

**Figure 3:**
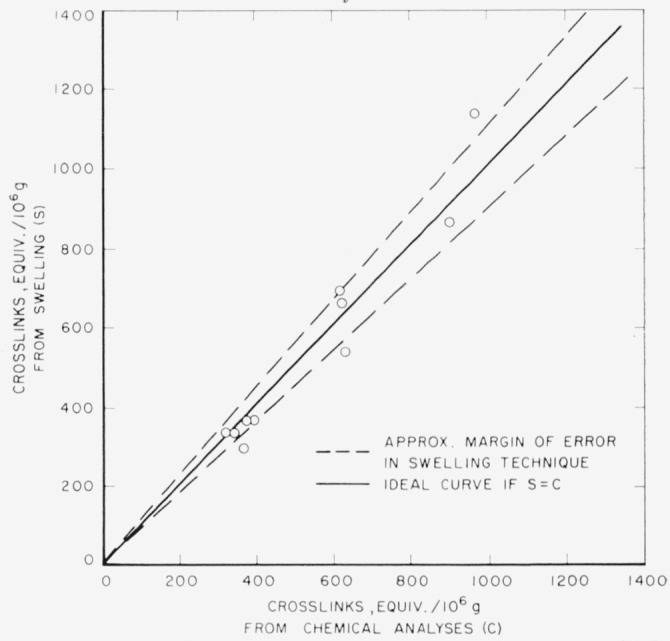
Correlation between equivalent number of crosslinks calculated from chemical analyses and swelling data.

Swelling measurements were carried out with *m*-cresol as the swelling agent at 25°C. Figure 1 illustrates the relationship between the calculated ${\bar{M}}_{c}$ (average molecular weight between crosslinks) and the experimentally determined *q_m_* (equilibrium swelling volume ratio) values. The values for ${\bar{M}}_{c}$ were calculated by using eq(9b), above, where χ_1_ = − 0.38 which value was obtained from the literature [3]. Figure 2 shows the equivalent number of crosslinks (–CH_2_–) between adjacent amide nitrogen atoms, calculated from chemical analyses and from swelling measurements, respectively, and their relationships to the percent formaldehyde. There is a good agreement between the results obtained from swelling measurements and chemical analyses, at least up to 1,000 equivalents of crosslinks per 10^6^ g of polymer (or 3 percent formaldehyde). This is further illustrated by ligure 3 in which the equivalent number of crosslinkages calculated from swelling data is plotted against those calculated from chemical analyses [6], The approximate margin of error, also indicated in figure 3, arises from the visual limitations of measuring the dimensions of the swollen fibers from photomicrographs. That equilibrium swelling was achieved within 30 min was established from the fact that the *q_m_* values showed no further changes beyond 30 min of swelling.

This good agreement indicates that the Flory- Rehner theory can be extended to this anisotropic system and suggests its possible general application to some anisotropic networks.

It is not yet clear to what extent it is possible to apply a modified Gaussian theory to an anisotropic network. However, the good agreement here with experimental data is encouraging. Current discussions on the theory of network structures [7, 8, 9] indicate that a rigorous analysis of this very complex problem is necessary and the present work suggests the desirability of further experimental data on numerous other polymer systems.

## Footnotes

1 Figures in brackets indicate the literature references at the end of this paper
